# Global Alliance for Genomics and Health Meets Bioconductor: Toward Reproducible and Agile Cancer Genomics at Cloud Scale

**DOI:** 10.1200/CCI.19.00111

**Published:** 2020-05-26

**Authors:** Vincent J. Carey, Marcel Ramos, Benjamin J. Stubbs, Shweta Gopaulakrishnan, Sehyun Oh, Nitesh Turaga, Levi Waldron, Martin Morgan

**Affiliations:** ^1^ Channing Division of Network Medicine, Brigham and Women’s Hospital, Harvard Medical School, Boston, MA; ^2^ Graduate School of Public Health and Health Policy, City University of New York, New York, NY; ^3^ Roswell Park Comprehensive Cancer Center, Buffalo, NY

## Abstract

**PURPOSE:**

Institutional efforts toward the democratization of cloud-scale data and analysis methods for cancer genomics are proceeding rapidly. As part of this effort, we bridge two major bioinformatic initiatives: the Global Alliance for Genomics and Health (GA4GH) and Bioconductor.

**METHODS:**

We describe in detail a use case in pancancer transcriptomics conducted by blending implementations of the GA4GH Workflow Execution Services and Tool Registry Service concepts with the Bioconductor curatedTCGAData and BiocOncoTK packages.

**RESULTS:**

We carried out the analysis with a formally archived workflow and container at dockstore.org and a workspace and notebook at app.terra.bio. The analysis identified relationships between microsatellite instability and biomarkers of immune dysregulation at a finer level of granularity than previously reported. Our use of standard approaches to containerization and workflow programming allows this analysis to be replicated and extended.

**CONCLUSION:**

Experimental use of dockstore.org and app.terra.bio in concert with Bioconductor enabled novel statistical analysis of large genomic projects without the need for local supercomputing resources but involved challenges related to container design, script archiving, and unit testing. Best practices and cost/benefit metrics for the management and analysis of globally federated genomic data and annotation are evolving. The creation and execution of use cases like the one reported here will be helpful in the development and comparison of approaches to federated data/analysis systems in cancer genomics.

## INTRODUCTION

Broadly stated, the computational initiatives of the Global Alliance for Genomics and Health (GA4GH) concern improvements in the efficiency of data management and analysis at a global level.^1^ Consortium-generated genome-scale data sets should be uniformly and securely managed in globally accessible systems, and analytic tools should be deployed on the data within these systems, minimizing investigator efforts devoted to data downloads and system configuration and eliminating the need for local high-performance computing infrastructure. The potential for federated data management and computation implicit in this vision is in contrast to the highly distributed, localized nature of bioinformatic method development and analysis prevalent to date.

**CONTEXT**

**Key Objective**
How can new concepts emerging from the Global Alliance for Genomics and Health help cancer scientists and clinicians do their work?
**Knowledge Generated**
New protocols for tool registration and workflow execution can be combined with easy-to-use open-source software and interactive notebook systems to broaden our knowledge of the impacts of tumor mutation profiles on gene expression. When mutation profiles are viewed along a spectrum, we can identify situations in which gene expression varies smoothly with a measure of mutation load.
**Relevance**
As genome sequencing costs decline, clinics will have access to whole-genome sequences, the interpretation of which will have implications for therapeutic choices. Informatic tools that integrate expression and genotype can be made easy to use, and with cloud computing, reliable and rapid interpretation of complex genomic data can be achieved.

In this article, we examine an approach to combining the GA4GH Tool Registry Services (TRS) and Workflow Execution Services (WES) concepts, as implemented in dockstore.org^2^ and the Broad Institute Cromwell workflow execution engine, with data, annotation, and software resources and practices developed in the Bioconductor project.^3,4^ The goal of an agile bioinformatic resource ecosystem requires principles of resource distribution and management that are coming into focus as new resources are brought to bear on problems of increasing size and importance. In this context, resources include data, annotation, software, documentation, analysis environments, and architectural materials related to overall system function, security, and evolution. We illustrate GA4GH-Bioconductor blending for working bioinformaticians by stepping through a problem in pancancer transcriptomics, using standard approaches to defining and distributing a container, and carrying out the analysis in the Broad Institute Terra computing environment.

## METHODS

### Use Case Definition

**FIG 1.**
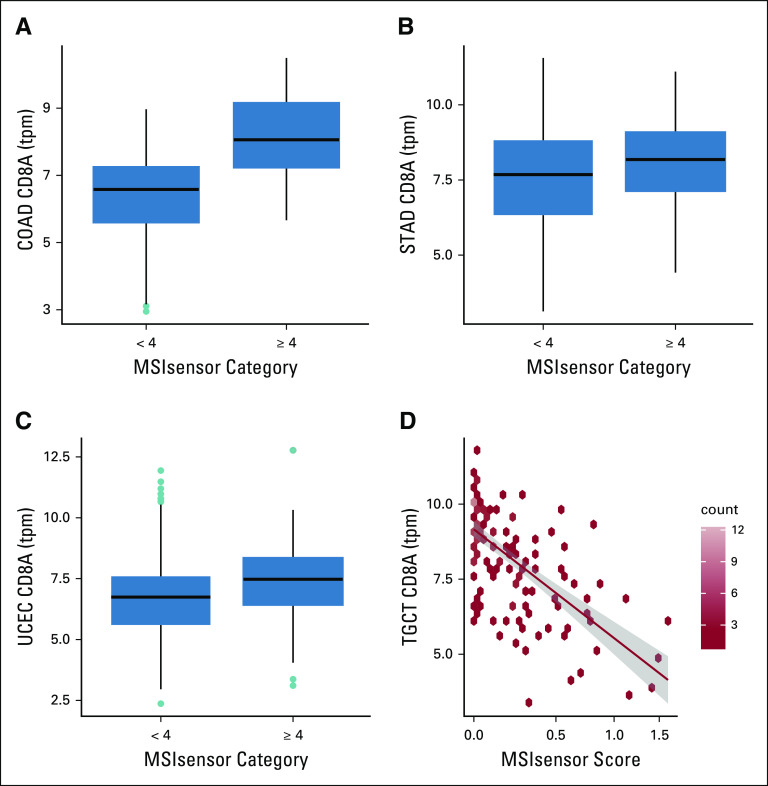
Illustration of two approaches to assessing tumor type–specific relationships between microsatellite instability and gene expression. (A-C) Qualitative reproduction of findings in Figure 5C of Bailey et al^5^: (A) colon adenocarcinoma (COAD), (B) stomach adenocarcinoma (STAD), and (C) uterine corpus endometrial carcinoma (UCEC). (D) Robust linear regression fit of mean expression of CD8A as a function of MSIsensor score as estimated for every The Cancer Genome Atlas tumor in Ding et al.^11^ TGCT, testicular germ cell tumor; tpm, transcripts per million.

Systematic associations between microsatellite instability (MSI) and expression of biomarkers of immune activation have been repeatedly reported, as illustrated in Figure 5C of the 2018 pancancer analysis of driver mutations by Bailey et al.^5^ These authors used The Cancer Genome Atlas (TCGA) expression data on tumors from colon or rectum, stomach, and uterine corpus, comparing scatterplots of expression of CD8A, CD8B, CD274, PDCD1, and PDCD1LG2 across strata defined by the MSIsensor measure of MSI,^6^ with the threshold set at a value of 4. Figures 1A to 1C show boxplots of CD8A (selected for reference from the panel of five genes in Bailey et al^5^) distributions, separating colon from rectal tumors. Our use case was defined as follows: expand on the analysis of Bailey et al^5^ across all TCGA tumor types, with the aim of establishing false discovery rates (FDRs) for hypotheses asserting a linear trend between the MSIsensor measure of MSI and expression levels of members of a freely chosen set of genes.

An illustration of the trend analysis is provided in Figure 1D, in which a robust linear regression line is superimposed on the scatterplot relating MSIsensor score to average expression of CD8A in testicular germ cell cancer (TGCT). The FDRs will be computed, adjusting for batch effects by allowing a fixed effect for RNA sequencing (RNA-seq) assay plate in the tumor type–specific regression models. The gene set that will be analyzed for all tumors is composed of 21 genes. The set is made up of 10 prognostically adverse and 10 prognostically favorable genes, as enumerated by Gentles et al^7^ in 2015, along with CD8A.

### Computational Approach

**FIG 2.**
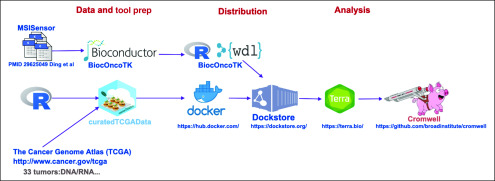
High-level overview of components of the analysis. R/Bioconductor is used to curate the microsatellite instability and expression data from The Cancer Genome Atlas and manage source code composed in R and Workflow Description Language (WDL) to specify the analysis. A Docker container is used to collect in a fully reproducible way all software infrastructure needed to execute the analysis. Dockstore is used to register code and container for durable public access and manage the conveyance of the workflow to the Terra platform, where the Cromwell workflow execution service manages the creation of the computational and storage environments where the analysis is carried out.

Figure 2 provides an overview of the components of the approach to this analysis.

#### Data resources.

We use Bioconductor 3.10 in R version 3.6.0 to obtain TCGA expression measures via the curatedTCGAData package.^8^ The measures denoted RNASeq2GeneNorm are extracted for each tumor type. These measures are upper-quartile normalized RSEM^9^ TPM gene expression values; in the analyses to be reported, we add 1 to each value and transform using base 2 logarithms. MSIsensor scores for 10,783 TCGA samples are available in the dingMSI data element of the BiocOncoTK package.^10^ These values are derived from Table S5 of the 2018 report by Ding et al.^11^

#### Containerization of software infrastructure and data.

The Bioconductor project manages Docker containers that provide complete Linux infrastructure to support installation and use of > 1,700 Bioconductor software packages and all their dependencies. We produced a container that includes the curatedTCGAData and BiocOncoTK packages and then executed code to retrieve and serialize curated versions of the expression and phenotype data for 33 TCGA tumors within the container. These serialized per-tumor MultiAssayExperiment instances are managed by facilities of the Bioconductor ExperimentHub package.^12^ The hub defined by this package combines an SQLite database of metadata about all hub components with a collection of file references to serialized data instances or remote resources. Requests for resources optionally include verification steps to determine whether a given resource in the hub requires updating. We chose to include the serialized TCGA data within the container to achieve persistence of this data image; the resulting container has a size of 4 GB. On a MacBook Air (2017 model), the container provides an R session within 5 seconds and delivers in-memory random access to all TCGA *BRCA* expression and clinical data within 30 seconds.

#### Workflow programming and component unit tests.

Two programs in the Workflow Description Language (WDL)^13^ are present in the inst/scripts/msireg folder of version 1.5.3 of the Bioconductor BiocOncoTK package. The GitHub repository for this package^14^ is used to register the WDL programs in dockstore.org. The workflow programs specify, using the scatter command, that computations for each gene in a gene list are to be run in parallel and that gene-specific computations for each tumor in TCGA are likewise run in parallel. The current version of WDL does not support nested scatter commands, so the tumor-level computations must be expressed in an external program that is imported to the program for gene-level computations. The WDL programs specify the desired hardware configurations in terms of the Docker container supplying system resources, boot and user disk sizes, volume of RAM, and maximum number of retries for failed tasks. These programs drive the data acquisition and analysis by calling R scripts in the BiocOncoTK package that are mirrored to globally accessible Google Storage buckets. These scripts make use of curatedTCGAData, BiocOncoTK, and ExperimentHub packages. Unit tests for the scripts are present in the BiocOncoTK package, and these tests are run nightly to verify that any changes to these packages in Bioconductor do not adversely affect this analysis.

#### Workflow execution.

Version 45 of the Cromwell WDL execution engine is available for use at app.terra.bio. Upon publishing the workflow at dockstore.org, a user can activate a control labeled Run in Terra. The browser is then directed to define a Terra workspace in which the workflow can be inserted. The user is prompted to specify inputs (values for any variables that do not have bindings in the WDL) and start the job. The Terra system provides basic facilities for monitoring job progress, and final results are delivered to a workspace-specific Google Storage bucket.

#### Analysis notebook.

**FIG 3.**
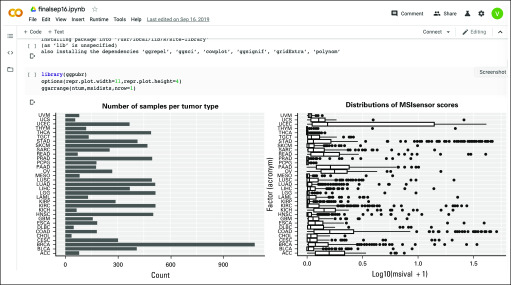
Screenshot of a segment of the Jupyter notebook recording exploratory and inferential computations for the pancancer transcriptomic use case. The code segments address package attachment and installation and production of graphics depicting sample sizes and distributions of MSIsensor scores for all tumor types in The Cancer Genome Atlas.

All computations related to data preparation, inference, and visualization are recorded and replayable in a Terra Jupyter notebook. Figure 3 provides a screenshot of this notebook, illustrating how code, narrative, and data visualizations are sequenced in interactive exploration.

### Statistical Methods

We evaluated the relationship between MSIsensor scores and expression of selected genes in two ways. We used two-sample *t* tests comparing samples with MSIsensor scores above and below the threshold of 4. We computed tests for trend between log(MSIsensor score + 1) and gene expression, allowing a fixed effect for assay plate, with linear regression. FDRs for these tests were computed using the method of Benjamini and Hochberg.^15^

## RESULTS

**FIG 4.**
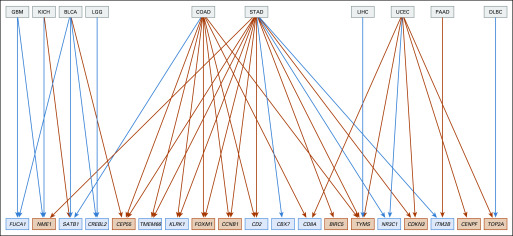
Existence and direction of significant associations (false discovery rate < 5%) between MSIsensor score > 4 and mean expression of selected genes for selected tumor types. Gene symbols in light blue boxes are described as prognostically favorable in Gentles et al^7^; symbols in orange boxes are prognostically unfavorable. A blue line between tumor type and gene indicates that MSIsensor scores > 4 associated with that tumor type are associated with decreased expression of the target gene (orange line, increased expression). BLCA, bladder urothelial carcinoma; DLBC, lymphoid neoplasm diffuse large B-cell lymphoma; COAD, colon adenocarcinoma; GBM, glioblastoma multiforme; KICH, kidney chromophobe; LIHC, liver hepatocellular carcinoma; LGG, brain lower-grade glioma; PAAD, pancreatic adenocarcinoma; STAD, stomach adenocarcinoma; UCEC, uterine corpus endometrial carcinoma.

**FIG 5.**
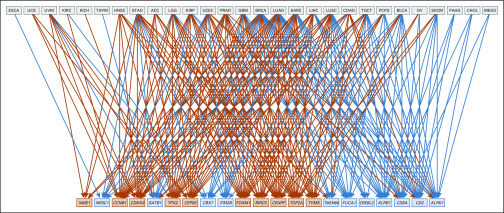
Bipartite graph presenting tumor type–gene pairs exhibiting significant linear association between log-transformed estimated MSIsensor score and log-transformed gene expression. Gene symbols in light blue boxes are described as prognostically favorable in Gentles et al^7^; symbols in orange boxes are prognostically unfavorable. A blue line between tumor type and gene indicates that MSIsensor scores associated with that tumor type are approximately linearly associated with decreased expression of the target gene (orange line, increased expression). ACC, adrenocortical carcinoma; BLCA, bladder urothelial carcinoma; BRCA, breast invasive carcinoma; CHOL, cholangiocarcinoma; COAD, colon adenocarcinoma; ESCA, esophageal carcinoma; GBM, glioblastoma multiforme; HNSC, head and neck squamous cell carcinoma; KICH, kidney chromophobe; KIRC, kidney renal clear cell carcinoma; KIRP, kidney renal papillary cell carcinoma; LIHC, liver hepatocellular carcinoma; LGG, brain lower-grade glioma; LUAD, lung adenocarcinoma; LUSC, lung squamous cell carcinoma; MESO, mesothelioma; OV, ovarian serous cystadenocarcinoma; PAAD, pancreatic adenocarcinoma; PCPG, pheochromocytoma and paraganglioma; PRAD, prostate adenocarcinoma; SARC, sarcoma; SKCM, skin cutaneous melanoma; STAD, stomach adenocarcinoma; TGCT, testicular germ cell tumors; THYM, thymoma; UCEC, uterine corpus endometrial carcinoma; UCS, uterine carcinosarcoma; UVM, uveal melanoma.

The WDL workflow was completed in Terra in 33 minutes. Downstream computations in the Jupyter notebook produced Figures 1, 4, and 5 in < 1 minute. The estimated Google Cloud Platform charge for the analysis reported here is $10.00, exclusive of resources consumed to construct the Docker container.

There are 693 (21 × 33) tumor type by gene combinations to be evaluated. Figures 1A to 1C present simple stratified comparisons, with the MSIsensor score threshold set at 4. Figure 1D unpacks the data on TGCT, for which all MSIsensor scores were < 4. The robust linear regression fit indicates that average CD8 expression decays monotonically with increasing MSIsensor scores. This finding is presented cautiously in light of the remark by Bailey et al that MSIsensor scores “below 4 cannot distinguish reliably between MSI-Low and MSS.”^5(pe6)^

When the 693 stratified comparisons are conducted using the Welch *t* test, 40 tumor type–gene pairs provide evidence of a relationship between MSI and expression with Benjamini-Hochberg FDR estimates of ≤ 5%. The 40 significant findings are depicted in Figure 4. An orange line joining a tumor to a gene indicates that for that tumor type, MSIsensor score > 4 is associated with higher expression of the gene (blue line, lower expression). Gene symbols in orange boxes have higher expression levels associated with unfavorable prognosis (light blue box, favorable prognosis), according to Gentles et al.^7^

Figure 5 presents plate-adjusted linear regression tests of association between MSIsensor scores and gene expression. Of the 693 tumor type–gene pairs evaluated, 246 exhibited significant trend tests (FDR < 5%) in the presence of adjustment for plate effects. The layout of the graph aims to minimize edge crossings. When MSI associated with a given tumor type is linked to a prognostically unfavorable gene (symbol in orange box), the association is always positive (134 of 134 significant pairs). When the link is to a prognostically favorable gene, the association is almost always negative (101 of 112 significant pairs).

We conclude that there is a prima facie case for considering the full spectrum of MSIsensor measures of MSI as a component of genomic analysis of tumor progression and treatment response, recognizing that the data configurations for different tumor types will often suggest analyses that are more detailed than the linear regressions applied uniformly in this use case. Further work to accommodate bimodality and heteroskedasticity in expression and accommodate sparsity in distribution of MSIsensor scores will lead to more secure inferences.

## DISCUSSION

This analysis was conceived as a vehicle for exploring a number of cross-cutting concerns in the democratization of genome-scale data and analysis. We tackled a problem of moderate scale, surveying processed RNA-seq expression data for 33 TCGA tumor types. We aimed to create a scalable, reproducible, and extensible analysis to address a relatively simple question concerning associations between MSI measures and gene expression patterns within TCGA tumor types. There were three main phases of development.

In the first phase, we assessed the scope of software components and persistent data required to execute the use case and produced a DockerHub-registered container provisioned to carry out the planned analysis. We explicitly incorporated all RNA-seq expression data for all TCGA tumors in this container.

In the second phase, we composed R scripts, and WDL workflows based on these scripts, to filter TCGA expression data managed in the Bioconductor ExperimentHub via the curatedTCGAData package. The scripts and WDL programs were added to the Bioconductor BiocOncoTK package and archived on GitHub. This archive was registered in dockstore.org and made available for use in the Broad Institute Terra environment via the Dockstore Application Programming Interface. A thorough tutorial on using Dockstore for general workflow management and publication is provided in the video at https://youtu.be/RYHUX9jGx24. The filtering of TCGA data and merging of MSIsensor scores were carried out with the Cromwell workflow execution engine in Terra. This produced an R data frame with 200,193 rows (21 rows for each of the 9533 primary tumor samples) in a Google Storage bucket.

The third phase of work involved interacting with this data frame in a Jupyter notebook within Terra to explore the data and modeling concepts (Fig 1) and derive the bipartite graphs (Figs 4 and 5). These interactions are recorded in a publicly accessible Google Colaboratory notebook. Figure 3 provides a screenshot of part of this notebook.

This analysis could have been carried out completely in R with Bioconductor or in any resource supporting statistical analysis of TCGA data, such as any of the Cancer Genomics Cloud projects.^16^ Adoption of the container plus workflow protocol of dockstore.org provided convenient access to Google Compute Platform facilities mediated through the Broad Institute app.terra.bio. The environment configuration is specified in WDL, and machines are started and stopped as required by workflow tasks. The workflow is parameterized by WDL inputs, in this case the sets of tumors and genes. Recomputing the workflow for different inputs is a matter of modifying lists of symbols and pressing a button.

The conveniences of dockstore plus Terra for expressing and executing complex analytic workflows, fully reproducibly, are compelling. We conclude with a consideration of certain aspects of the Bioconductor software/analysis/data ecosystem that will add value to the container/workflow-oriented framework as it evolves.

Bioconductor is a self-sufficient framework for conducting genome-scale data analyses of many kinds. Infrastructure for genome representation and annotation is substantial and mature, packages for state-of-the-art statistical methods are constantly being added, and various approaches to scalable use of memory and parallel computing facilities are readily available. The growth of Bioconductor and its communities of users and developers is in part a result of this capacity for creating self-sufficient, stable, well-provisioned environments for investigators and laboratories. Reliability of the system is rooted in high-frequency continuous integration testing of all packages. Reliability of specific computing activities is rooted in part in the commitment to protocols for software packaging, documentation and testing, emphasizing self-describing data structures for annotation (always documenting the reference build to which any genomic coordinate refers), and experiments (tightly binding metadata and sample data to assay data). Insofar as the development of components of the container plus workflow framework take us outside standard practices underlying the Bioconductor ecosystem, there are risks of systematic loss of key sources of reliability.

To partly mitigate these risks in this example, we defined unit tests for the R scripts that manipulate TCGA data in our workflow. These tests are now part of the BiocOncoTK package and thus are run every night. Should any aspect of infrastructure or dependent software or data change in a way that alters the outputs of these scripts, the BiocOncoTK check will fail, and its developer will be prompted to undertake repair of the associated code. In principle, this practice of incorporating workflow components into continuously integrated packages can extend to incorporating and testing the workflow itself. A basic question is how this can be conducted independently of the container that workflow used in its primary application. By design, the container plus workflow registered on dockstore.org will have predictable behaviors and thus would not seem to need continuous testing. But if the workflow is to have value in reuse over time, its components should be regularly tested in the evolving ecosystem of which it is a part. The testing protocol implemented for this workflow is limited, and further research and development on the general topic of cost effectiveness of testing workflow accuracy and durability are warranted.

In summary, new approaches to management and analysis of globally federated genomic data and annotation provide new opportunities for bioinformaticians. We describe experimentation with the GA4GH TRS and WES frameworks, with deployments at dockstore.org and execution in the Broad Institute app.terra.bio. Future work will demonstrate how to relax platform dependence of the solution to the use case presented here, abstracting from the specific data model and execution system used.

The objective of democratizing cloud-scale data and analysis methods for human genomic research is coming into focus. Durable solutions must respect rapidity and disruptiveness of evolution in the technologies of biologic experimentation and computing. It is hoped that the creation and execution of use cases like the one reported here will be helpful in the comparison of different approaches to the use of containers and federated data/analysis systems in cancer genomics. Specifically, we believe that the tractability and reproducibility of clinical research efforts involving computational analysis of cutting-edge assays carried out on newly assembled cohorts will be greatly enhanced through use of the GA4GH-based concepts and tools outlined here.
